# Cancer risk in adrenalectomy: are adrenal lesions equal or more than 4 cm a contraindication for laparoscopy?

**DOI:** 10.1007/s00464-021-08380-7

**Published:** 2021-03-01

**Authors:** Andrea Balla, Diletta Corallino, Monica Ortenzi, Livia Palmieri, Francesca Meoli, Mario Guerrieri, Alessandro M. Paganini

**Affiliations:** 1 Department of General Surgery and Surgical Specialties “Paride Stefanini”, Sapienza University of Rome, Azienda Policlinico Umberto I, Viale del Policlinico 155, 00161 Rome, Italy; 2 Department of General Surgery, Università Politecnica delle Marche, Piazza Roma 22, 60121 Ancona, Italy

**Keywords:** Adrenal cancer risk, Adrenal tumors, Laparoscopic adrenalectomy (LA), Open adrenalectomy (OA), Adrenal lesion size 4 cm

## Abstract

**Background:**

Some authors consider adrenal lesions size of less than 4 cm as a positive cut-off limit to set the indications for minimally invasive surgery due to a lower risk of malignancy. Aim of this study is to report the risk of cancer for adrenal lesions measuring 4 cm or more in diameter, assessed as benign at preoperative workup (primary outcome), and to evaluate the feasibility and safety of laparoscopic adrenalectomy (LA) in these cases (secondary outcome).

**Methods:**

From January 1994 to February 2019, 579 patients underwent adrenalectomy. Fifty patients with a preoperative diagnosis of primary adrenal cancer or metastases were excluded. The remaining 529 patients were included and divided in five subgroups based on adrenal lesion size at definitive histology: group A, 4–5.9 cm (137 patients); group B, 6–7.9 cm (64 patients); group C, 8–9.9 cm (13 patients); group D, ≥ 10 cm (11 patients); group E, < 4 cm (304 patients). Each group was further divided based on diagnosis of benign or malignant lesions at definitive histology.

**Results:**

Four (2.9%) malignant lesions were observed in group A, 5 (7.8%) in group B, 2 (15.4%) in Groups C and D (18.2%) and 13 (4.3%) in Group E. Comparing the cancer risk among the groups, no statistically significant differences were observed. Operative time increased with increasing lesion size. However, no statistically significant differences were observed between benign and malignant lesions in each group comparing operative time, conversion and complication rates, postoperative hospital stay and mortality rate.

**Conclusions:**

Adrenal lesions measuring 4 cm or more in diameter are not a contraindication for LA neither in terms of cancer risk nor of conversion and morbidity rates, even if the operative time increases with increasing adrenal lesion diameter. Further prospective studies with a larger number of patients are required to draw definitive conclusions.

Laparoscopic transperitoneal lateral adrenalectomy (LA) was first described by Gagner in 1992 [1]. Since then, several laparoscopic approaches have been proposed, including the anterior transperitoneal approach, with the submesocolic option for left sided lesions, and the retroperitoneal approach with patient in prone or lateral decubitus position [2–5]. Robotic adrenalectomy and single-incision laparoscopic surgery (SILS) approach have also been more recently proposed [4–8]. Although minimally invasive surgery (MIS) has become the gold standard for adrenal surgery, the superiority of one approach over another has not been demonstrated yet [9, 10].

Despite MIS is associated with better postoperative outcomes in terms of less pain, shorter hospital stay and faster functional recovery [11–14], as compared to open adrenalectomy (OA), whether large adrenal lesions should be managed by MIS is still debated [15–17]. MIS for large adrenal lesions has been reported to entail longer operative time, increased intraoperative blood loss and higher morbidity rate due to more difficult gland dissection [17, 18]. Moreover, the risk of adrenal malignancy has been reported to increase with increasing tumor size, but whether tumor size alone should be considered an absolute contraindication for MIS has yet to be defined [19, 20]. OA is generally preferred in case of lesions at high risk of malignancy, due to the increased risk of peritoneal spread of cancer cells during MIS and of local recurrences [21, 22]. Some authors consider adrenal lesions size of less than 4 cm as a positive cut-off limit to set the indications for MIS due to a lower risk of malignancy [19, 23–26].

The aim of this study is to report the risk of cancer in a consecutive series of patients with adrenal gland lesions measuring 4 cm or more in diameter that were assessed as benign at preoperative workup (primary outcome), and to evaluate the feasibility and safety of the laparoscopic approach in these cases (secondary outcome).

## Materials and methods

This study is a retrospective analysis of prospectively collected data, approved by our Institutional review board. Informed consent was obtained from all participants.

From January 1994 to February 2019, 579 patients underwent surgery for adrenal gland disease in two centers (Department of General Surgery and Surgical Specialties "Paride Stefanini", Sapienza University of Rome and Department of General Surgery, Università Politecnica delle Marche, Ancona, Italy) that followed the same treatment protocol and used an identical surgical approach, as previously reported [4, 9, 10, 27].

**Fig. 1:**
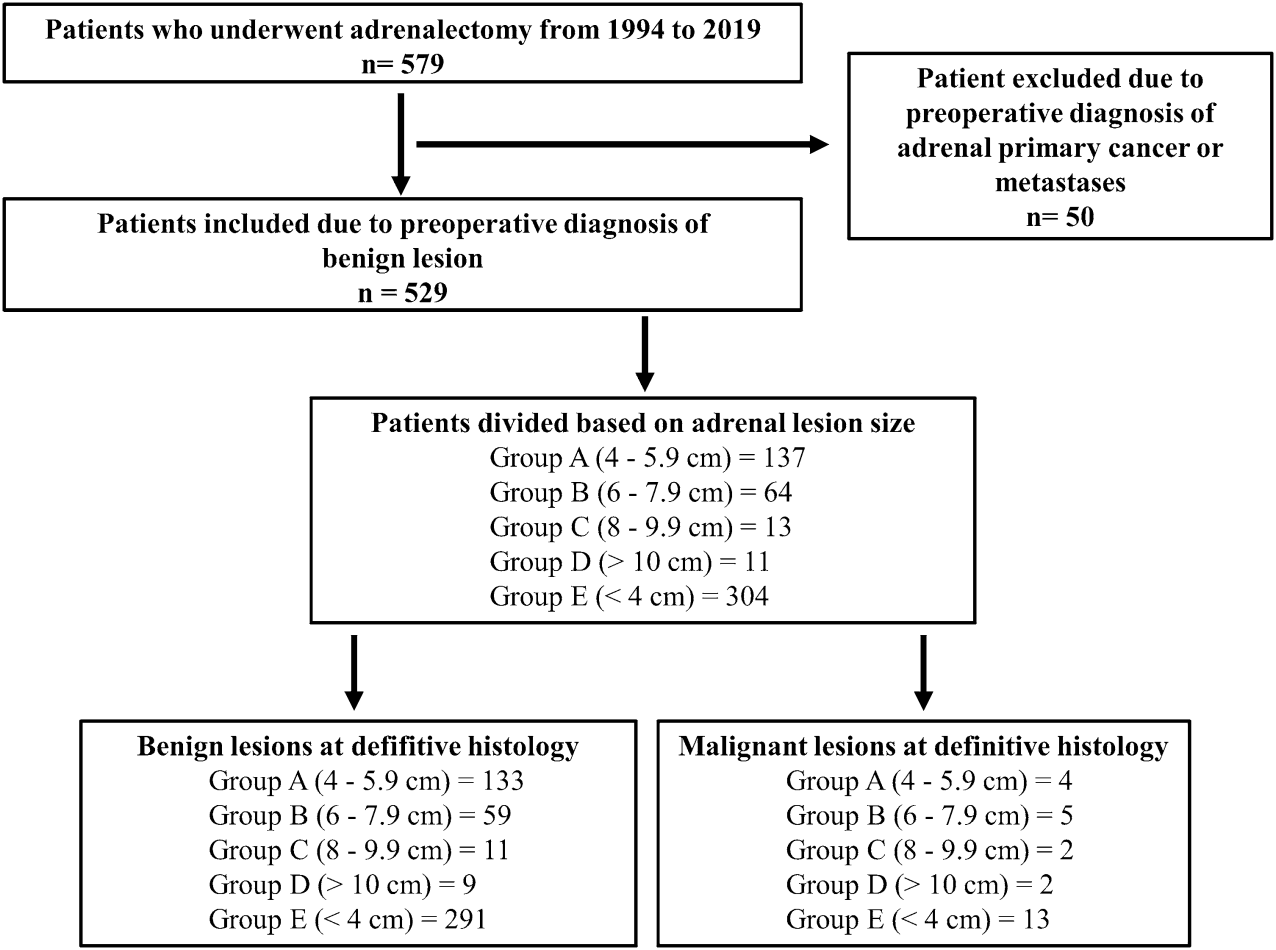
Patients selection

All patients were studied preoperatively with computed tomography (CT) scan and magnetic resonance imaging (MRI). On unenhanced CT scan, an attenuation higher than 10 Hounsfield Units (HU) was considered as suggestive for carcinoma [10]. On MRI, the diagnosis of adrenal carcinoma was made in case of heterogeneity on T1-weighted images with intermediate to high signal intensity [4, 10]. The diagnosis of Cushing’s and Conn’s syndrome and pheochromocytoma was made as previously described [4, 10]. The diagnosis of non-secreting adenoma was made in the absence of specific signs and/or symptoms of adrenal autonomous hormone secretion, abnormal hypothalamus–pituitary–adrenal axis tests and an imaging compatible with an adrenocortical lesion [4, 10]. Patients with a preoperative diagnosis of primary adrenal cancer or metastases were excluded from the present study (50 patients, 8.6%), leaving 529 patients (91.4%) to be included in the study (Fig. 1).

Out of 529 patients, 258 (48.8%), 249 (47.1%) and 22 (4.2%) underwent right, left or bilateral adrenalectomy, respectively. As previously reported, LA was performed mainly by the anterior approach with patients in supine position [4, 9, 10, 27]. In this series, the anterior transperitoneal approach was performed in 262 patients (49.6%), the anterior transperitoneal submesocolic approach in case of left adrenalectomy was performed in 244 patients (46.1%) and the lateral transperitoneal approach was performed in the remaining 23 patients (4.3%).

### Study design

Included patients were divided into five subgroups based on adrenal lesion size at definitive histology: group A, from 4 to 5.9 cm (137 patients); group B, from 6 to 7.9 cm (64 patients); group C, from 8 to 9.9 cm (13 patients); group D, ≥ 10 cm (11 patients); group E, < 4 cm (304 patients). Each group was further divided based on benign and malignant lesions at definitive histology (Fig. 1).

Indication to surgery, gender, age, body mass index (BMI), previous abdominal surgery, lesion side, surgical approach, associated procedures, conversion rate, operative time, intra and postoperative complications (graded according to the Clavien-Dindo Classification [28]), adrenal size on the specimen, definitive histology, hospital stay, 30-day mortality and oncological follow up data were collected in a Microsoft Excel program (Microsoft Corporation, Redmond, Washington, USA).

### Statistical analysis

Continuous variables are expressed as mean ± standard deviation (SD) while categorical variables as frequencies and percentages. The Mann Whitney *U* test and Fisher’s exact test were used for the comparison between groups of continuous and categorical variables, respectively. A *p* value lower than 0.05 was considered statistically significant. Statistical analyses were carried out with SPSS software 22.0 (SPSS Inc., Chicago, Illinois, USA).

## Results

Table 1 shows the cancer risk for each group: 4 (2.9%) malignant lesions were observed in group A, 5 (7.8%) in group B, 2 (15.4%) in Groups C and D (18.2%) and 13 (4.3%) in Group E. No statistically significant differences were observed comparing the risk of cancer among the five groups. Overall, the cancer risk for adrenal lesions measuring 4 cm or more in this series was 5.7%, on average. Table 2 reports the definitive histology of malignant lesions for each group.

**Table 1:** Cancer risk stratified based on adrenal lesion size

Group (cm)	Cancer risk, *n* (%)	*p* value
A (4–5.9)	4 (2.9)	vs B: 0.147 vs C: 0.086 vs D: 0.064 vs E: 0.601
B (6–7.9)	5 (7.8)	vs C: 0.336 vs D: 0.272 vs E: 0.216
C (8–9.9)	2 (15.4)	vs D: 1.000 vs E: 0.121
D (≥ 10)	2 (18.2)	vs E: 0.0908
E (< 4)	13 (4.3)	vs A + B + C + D: 0.542

**Table 2:** Definitive histology for each group of patients

Definitive histology	Groups				
	A *n* = 137	B *n* = 64	C *n* = 13	D *n* = 11	E *n* = 304
Primary adrenal lesion, *n* (%)					
Adrenal carcinoma	1 (0.7)	4 (6.3)	2 (15.4)	0	6 (2.0)
Pheochromocytoma	2 (1.5)	0	0	0	7 (2.3)
Adrenal metastases, *n* (%)					
Liposarcoma	0	0	0	2 (18.2)	0
Lung	1 (0.7)	0	0	0	0
Bladder	0	1 (1.6)	0	0	0
Total	4 (2.9)	5 (7.8)	2 (15.4)	2 (18.2)	13 (4.3)

In all 26 malignant lesions, capsule rupture with intraoperative tumor spillage did not occur. R0 resection was achieved in all patients.

Patients’ characteristics and surgical outcomes for each group, comparing benign versus malignant lesions, are shown in Tables 3, 4, 5, 6, 7. In Group A, B, C and D statistically significant differences between each variable were not observed (Tables 3, 4, 5, 6). In Group E the number of patients with Conn’s or Cushing’s syndrome and pheochromocytoma were significantly higher in patients with benign lesions (*p* = 0.003, *p* = 0.024, respectively) (Table 7).

**Table 3:** Patients’ characteristics and surgical outcomes in group A

Group (cm)	Indication to surgery	Benign lesions at preoperative workup		*p* value
		Benign lesions at definitive histology	Malignant lesions at definitive histology	
	*n* = 137 (25.9%)	*n* = 133 (97.1%)	*n* = 4 (2.9%)	
A	Conn’s–Cushing’s syndrome, *n* (%)	35 (26.3)	–	0.572
4–5.9	Adenoma	21 (15.8)	–	1.000
	Hyperplasia	14 (10.5)	–	1.000
	Pheochromocytoma, *n* (%)	34 (25.6)	1 (25)	1.000
	Other type of lesion, *n* (%)	64 (48.1)	3 (75)	0.359
	Myelolipoma	12 (9.0)	–	1.000
	Non-secreting adenoma	48 (36)	3 (75)	0.145
	Adrenal cyst	3 (2.3)	–	1.000
	Angiomyolipoma	1 (0.8)	–	1.000
	Hormonal activity (active/inactive), *n* (%)	68 (51.1)/65 (48.9)	1 (25)/3 (75)	0.366
	Sex ratio (men: women)	52:81	3:1	0.303
	Mean age ± SD, years (range)	54.8 ± 15 (22–84)	56.5 ± 22.7 (25–78)	0.636
	Mean body mass index ± SD, kg/m^2^ (range)	27.5 ± 5.4 (18–48)	26 ± 5.5 (16.8–29)	0.457
	Previous abdominal surgery, *n* (%)	89 (66.9)	1 (25)	0.117
	Lesion side, *n* (%)			
	Right	77 (57.9)	2 (50)	1.000
	Left	51 (38.3)	2 (50)	0.640
	Bilateral	5 (3.8)	–	1.000
	Surgical approach, *n* (%)			
	Laparoscopic	132 (99.2)	3 (75)	0.058
	Open	1 (0.8)	1 (25)	
	Associated procedures, *n* (%)	2 (1.5)	1 (25)	0.086
	Conversion rate, *n* (%)	7 (5.3)	–	1.000
	Adhesions for previous surgery	2 (1.5)	–	1.000
	Adhesion to pancreas	1 (0.8)	–	1.000
	Adhesion to liver	1 (0.8)	–	1.000
	Bleeding	2 (1.5)	–	1.000
	Ureteral injury	1 (0.8)	–	1.000
	Mean operative time ± SD, min (range)	99 ± 54.1 (30–360)	87.5 ± 10.8 (80–105)	0.654
	Postoperative complications, *n* (%, Clavien-Dindo classification, grade)	17 (12.8)	–	1.000
	Surgical complications	11 (8.3)	–	1.000
	Ileus	1 (0.8, I)	–	1.000
	Acute urinary retention	1 (0.8, I)	–	1.000
	Wound infection	2 (1.5, II)	–	1.000
	Anemia	3 (2.3, II)	–	1.000
	Fever	3 (2.3, II)	–	1.000
	Hemoperitoneum	1 (0.8, III-b)	–	1.000
	Medical complications	6 (4.5)	–	1.000
	Pneumonia	1 (0.8, I)	–	1.000
	Atrial fibrillation	1 (0.8, II)	–	1.000
	Respiratory failure	2 (1.5, II)	–	1.000
	Acute myocardial infarction	1 (0.8, V)	–	1.000
	Ventricular fibrillation	1 (0.8, V)	–	1.000
	Mean lesion size at definitive histology ± SD, cm (range)	4.3 ± 0.6 (3–5.7)	4.6 ± 0.6 (4–5.5)	0.260
	Mean hospital stay ± SD, days (range)	4.4 ± 2.8 (2–19)	4.5 ± 1.7 (3–7)	0.541
	Mortality, *n* (%)	2 (1.5)	–	1.000

*Group A* patients with adrenal lesions size between 4 and 5.9 cm, *SD* standard deviation

**Table 4:** Patients’ characteristics and surgical outcomes in group B

Group (cm)	Indication to surgery	Benign lesions at preoperative workup		*p* value
		Benign lesions at definitive histology	Malignant lesions at definitive histology	
	*n* = 64 (12.0%)	*n* = 59 (92.2%)	*n* = 5 (7.8%)	
B	Conn’s–Cushing’s syndrome, *n* (%)	12 (20.3)	2 (40)	0.299
6–7.9	Adenoma	5 (8.5)	2 (40)	0.088
	Hyperplasia	7 (11.9)	–	1.000
	Pheochromocytoma, *n* (%)	21(35.6)	–	0.163
	Other type of lesion, *n* (%)	26 (44.1)	3 (60)	0.652
	Myelolipoma	3 (5.1)	–	1.000
	Non-secreting adenoma	22 (37.3)	3 (60)	0.371
	Adrenal cyst	1 (1.7)	–	1.000
	Hormonal activity (active/inactive), *n* (%)	34 (57.6)/25 (42.4)	2 (40)/3 (60)	0.646
	Sex ratio (men: women)	32:27	2:3	0.659
	Mean age ± SD, years (range)	52.9 ± 12.7 (23–77)	56.2 ± 19.9 (21–69)	0.369
	Mean body mass index ± SD, kg/m^2^ (range)	26.7 ± 4.6 (19–39)	23.7 ± 2.1 (21–26)	0.114
	Previous abdominal surgery, *n* (%)	20 (33.9)	3 (60)	0.341
	Lesion side, *n* (%)			
	Right	28 (47.5)	1 (20)	0.367
	Left	28 (47.5)	4 (80)	0.355
	Bilateral	3 (5.1)	–	1.000
	Surgical approach, *n* (%)			
	Laparoscopic	57 (96.6)	5 (100)	1.000
	Open	2 (3.4)	–	
	Associated procedures, *n* (%)	2 (3.4)	–	1.000
	Conversion rate, *n* (%)	5 (8.5)	–	1.000
	Bleeding	2 (3.4)	–	1.000
	Absence of a cleavage plan	2 (3.4)	–	1.000
	Retrocaval mass growth	1 (1.7)	–	1.000
	Mean operative time ± SD, min (range)	111.5 ± 72.9 (30–360)	75 ± 40.7 (50–75)	0.602
	Postoperative complications, *n* (%, Clavien-Dindo classification, grade)	3 (5.1)	–	1.000
	Anemia	1 (1.7, II)	–	1.000
	Fever	2 (3.4, II)	–	1.000
	Mean lesion size at definitive histology ± SD, cm (range)	6.4 ± 0.5 (6–7.8)	7 ± 0.7 (6–7.5)	0.055
	Mean hospital stay ± SD, days (range)	4.63 ± 2.8 (2–15)	3 ± 1.22 (2–5)	0.203
	Mortality, *n* (%)	–	–	1.000

*Group B* patients with adrenal lesions size between 6 and 7.9 cm, *SD* standard deviation

**Table 5:** Patients’ characteristics and surgical outcomes in group C

Group (cm)	Indication to surgery	Benign lesions at preoperative workup		*p* value
		Benign lesions at definitive histology	Malignant lesions at definitive histology	
	*n* = 13 (2.5%)	*n* = 11 (84.6%)	*n* = 2 (15.4%)	
C	Conn’s–Cushing’s syndrome, *n* (%)	3 (27.3)	1 (50)	1.000
8–9.9	Adenoma	1 (9.1)	–	1.000
	Hyperplasia	2 (18.2)	–	1.000
	Pheochromocytoma, *n* (%)	2 (18.2)	–	1.000
	Other type of lesion, *n* (%)	6 (54.5)	1(50)	1.000
	Myelolipoma	1 (9.1)	–	1.000
	Non-secreting adenoma	4 (36.4)	1(50)	1.000
	Adrenal cyst	1 (9.1)	–	1.000
	Hormonal activity (active/inactive), *n* (%)	6 (54.5)/5 (45.5)	1 (50)/1 (50)	1.000
	Sex ratio (men: women)	6:5	1:1	1.000
	Mean age ± SD, years (range)	51.4 ± 16.8 (22–77)	24.5 ± 6.4 (20–29)	0.051
	Mean body mass index ± SD, kg/m^2^ (range)	27.2 ± 8.1 (21–45)	21.6 ± 4.5 (18.4–24.8)	0.400
	Previous abdominal surgery, *n* (%)	3 (27.3)	1 (50)	1.000
	Lesion side, *n* (%)			
	Right	4 (36.4)	–	1.000
	Left	6 (54.5)	2 (100)	0.487
	Bilateral	1 (9.1)	–	1.000
	Surgical approach, *n* (%)			
	Laparoscopic	9 (81.8)	1 (50)	0.423
	Open	2 (18.2)	1 (50)	
	Associated procedures, *n* (%)	–	–	1.000
	Conversion rate, *n* (%)	2 (18.2)	–	1.000
	Adhesions for previous surgery	1 (9.1)	–	1.000
	Mass size	1 (9.1)	–	1.000
	Mean operative time ± SD, min (range)	113 ± 55 (60–230)	90 ± 14.1 (80–100)	0.606
	Postoperative complications, *n* (%, Clavien-Dindo classification, grade)			
	Anemia	1 (9.1, II)	–	1.000
	Mean lesion size at definitive histology ± SD, cm (range)	8.4 ± 0.5 (8–9.3)	8.5 ± 0.7 (8–9)	0.923
	Mean hospital stay ± SD, days (range)	4.3 ± 2.6 (2–10)	4.5 ± 2.1 (3–6)	0.727
	Mortality, *n* (%)	–	–	1.000

*Group C* patients with adrenal lesions size between 8 and 9.9 cm, *SD* standard deviation

**Table 6:** Patients’ characteristics and surgical outcomes in group D

Group (cm)	Indication to surgery	Benign lesions at preoperative workup		*p* value
		Benign lesions at definitive histology	Malignant lesions at definitive histology	
	*n* = 11 (2.1%)	*n* = 9 (81.8%)	*n* = 2 (18.2%)	
D	Conn’s–Cushing’s syndrome, *n* (%)	4 (44.4)	–	0.491
≥ 10	Adenoma	2 (22.2)	–	1.000
	Hyperplasia	2 (22.2)	–	1.000
	Pheochromocytoma, *n* (%)	1 (11.1)	–	1.000
	Other type of lesion, *n* (%)	4 (44.4)	–	0.491
	Myelolipoma	2 (22.2)	–	1.000
	Non-secreting adenoma	1 (11.1)	2 (100)	0.055
	Adrenal cyst	1 (11.1)	–	1.000
	Hormonal activity (active/inactive), *n* (%)	6 (66.7)/3 (33.3)	0/2 (100)	1.000
	Sex ratio (men: women)	6:3	2:0	1.000
	Mean age ± SD, years (range)	59 ± 13.6 (40–82)	69 ± 9.9 (62–76)	0.327
	Mean body mass index ± SD, kg/m^2^ (range)	32 ± 7.4 (23.8–43.6)	24.4 ± 2.2 (22.9–25.9)	0.286
	Previous abdominal surgery, *n* (%)	1 (11.1)	–	1.000
	Lesion side, *n* (%)			
	Right	2 (22.2)	2 (100)	0.109
	Left	7 (77.8)	–	0.109
	Surgical approach, *n* (%)			
	Laparoscopic	4 (44.4)	–	0.491
	Open	5 (55.6)	2 (100)	
	Associated procedures, *n* (%)	1 (11.1)	–	1.000
	Conversion rate, *n* (%)	1 (25)	–	1.000
	Adhesions to vena cava	1 (25)	–	1.000
	Mean operative time ± SD, min (range)	134.4 ± 59.9 (60–240)	112.5 ± 31.8 (90–135)	0.711
	Postoperative complications, *n* (%, Clavien-Dindo classification, grade)			
	Anemia	1 (11.1, II)	–	1.000
	Mean lesion size at definitive histology ± SD, cm (range)	11.4 ± 1.7 (10–15)	11 ± 1.4 (10–12)	0.909
	Mean hospital stay ± SD, days (range)	7.6 ± 5.5 (4–21)	8 ± 0 (8–8)	0.178
	Mortality, *n* (%)	–	–	1.000

*Group D* patients with adrenal lesions size of more than 10 cm, *SD* standard deviation

**Table 7:** Patients’ characteristics and surgical outcomes in group E

Group (cm)	Indication to surgery	Benign lesions at preoperative workup		*p* value
		Benign lesions at definitive histology	Malignant lesions at definitive histology	
	*n* = 304 (57.5%)	*n* = 291 (95.7%)	*n* = 13 (4.3%)	
E	Conn’s–Cushing’s syndrome, *n* (%)	171 (58.8)	2 (15.4)	**0.003**
< 4	Adenoma	134 (46.0)	2 (15.4)	**0.043**
	Hyperplasia	37 (12.7)	–	0.380
	Pheochromocytoma, *n* (%)	38 (13.0)	5 (38.5)	**0.024**
	Other type of lesion, *n* (%)	82 (28.2)	6 (46.2)	0.209
	Myelolipoma	7 (2.4)	–	1.000
	Non-secreting adenoma	73 (25.1)	6 (46.2)	0.108
	Adrenal cyst	1 (0.3)	–	1.000
	Angiomyolipoma	1 (0.3)	–	1.000
	Hormonal activity (active/inactive), *n* (%)	210 (72.2)/81 (27.8)	7 (53.8)/6 (46.2)	0.207
	Sex ratio (men: women)	114:177	6:7	0.773
	Mean age ± SD, years (range)	51.5 ± 14.1 (17–81)	55.4 ± 14.1 (42–72)	0.343
	Mean body mass index ± SD, kg/m^2^ (range)	27.4 ± 5.4 (17.5–47)	26.7 ± 3.9 (20–31.6)	0.918
	Previous abdominal surgery, *n* (%)	57 (19.6)	1 (7.7)	0.474
	Lesion side, *n* (%)			
	Right	136 (46.7)	6 (46.2)	1.000
	Left	142 (48.8)	7 (53.8)	1.000
	Bilateral	13 (4.5)	–	1.000
	Surgical approach, *n* (%)			
	Laparoscopic	284 (97.6)	13 (100)	1.000
	Open	7 (2.4)	–	1.000
	Associated procedures, *n* (%)	4 (1.4)	1 (7.7)	0.198
	Conversion rate, *n* (%)	9 (3.1)	2 (15.4)	0.075
	Bleeding	4 (1.4)	1 (7.7)	0.198
	Adhesions for previous surgery	2 (0.7)	–	1.000
	Absence of a cleavage plan	2 (0.7)	1 (7.7)	0.123
	Respiratory failure for pneumoperitoneum	1 (0.3)	–	1.000
	Mean operative time ± SD, min (range)	105.4 ± 50.8 (30–360)	111.9 ± 48.7 (50–111)	0.479
	Postoperative complications, *n* (%, Clavien-Dindo classification, grade)	17 (5.8)	–	1.000
	Surgical complications	14 (4.8)	–	1.000
	Ileus	1 (0.3, I)	–	1.000
	Wound infection	1 (0.3, I)	–	1.000
	Anemia	6 (2.1, II)	–	1.000
	Abdominal abscess	2 (0.7, II)	–	1.000
	Hemoperitoneum	2 (0.7, III-a)	–	1.000
	Colic fistula	1 (0.3, III-a)	–	1.000
	Chylous ascites	1 (0.3, III-a)	–	1.000
	Medical complications	3 (1.0)	–	1.000
	Pleural effusion	1 (0.3, I)	–	1.000
	Pneumonia	2 (0.7, II)	–	1.000
	Mean lesion size at definitive histology ± SD, cm (range)	2.4 ± 0.9 (0.7–5)	2.7 ± 1.0 (1–3.6)	0.169
	Mean hospital stay ± SD, days (range)	4.3 ± 3.5 (1–30)	5 ± 1.6 (3–8)	0.582
	Mortality, *n* (%)	–	–	1.000

Statistically significant differences in bold

*Group E* patients with adrenal lesions size of less than 4 cm, *SD* standard deviation

Comparing the incidence of secreting lesions between malignant and benign histology for each group statistically significant differences were not observed (Tables 3, 4, 5, 6, 7).

Overall, OA was performed in 21 patients (4%): 1 (0.2%) in group B, for the concomitant presence of an abdominal aortic aneurysm (benign lesion), 2 (0.4%) in group D, for adhesion due to previous surgery (benign lesion). For the remaining eighteen patients (3.4%), although no clear signs of malignancy on preoperative imaging were present, the surgeon’s choice was to perform OA due to lesion size and growth. Four of these patients (0.8%) turned out to be malignant lesions on definitive histology (Tables 3, 4, 5, 6, 7).

In group A, an associated surgical procedure was performed in two patients with benign adrenal lesions (two cholecystectomies for symptomatic gallstones) and in one patient with a malignant lesion (left nephrectomy for the intraoperative finding of lack of a cleavage plane with the left kidney) (Table 3). In group B, two patients with benign lesions underwent concomitant cholecystectomy for gallstones, associated in one of them with laparoscopic choledochotomy and common bile duct exploration for removal of ductal stones (this patient underwent bilateral adrenalectomy) (Table 4). In group D, one patient with a benign adrenal lesion underwent concomitant cholecystectomy (Table 6). Finally, in group E, four patients with benign lesions underwent cholecystectomy (three patients) and umbilical hernia repair (one patient), while one patient with a malignant lesion underwent associated right nephrectomy. In this case the procedure was converted to open surgery due to intraoperative finding of absence of a cleavage plane (Table 7).

No statistically significant differences were observed between benign and malignant lesions in each group related to operative time, conversion and complication rates, postoperative hospital stay and mortality rate (Tables 3, 4, 5, 6, 7).

During the follow up period, local recurrences were not observed. Three patients (11.5%) with malignant lesions at definitive histology died from disease progression: one patient in group B with diagnosis of adrenal carcinoma measuring 7.5 cm who underwent LA, one patient in group C with diagnosis of adrenal carcinoma measuring 9 cm who underwent LA and converted to OA due to mass size and one patient in group D with diagnosis of liposarcoma measuring 10 cm who underwent OA due to suspicion of possible intraoperative technical problems.

## Discussion

Aim of this study was to investigate if LA for adrenal lesions measuring 4 cm or more in diameter, and considered as benign preoperatively, is oncologically and surgically safe, in order to avoid OA and to reserve this approach as a possible alternative if loco-regional conditions do not allow an oncologically and surgically safe LA.

For this purpose, the patients were stratified in five subgroups based on the adrenal lesion size, and then in each subgroup a comparison was made between benign and malignant lesions at definitive histology. Group E served as the control group. As expected, and as reported in literature [19, 23–26], the adrenal cancer risk that was observed in this study increased with increasing lesion size, although this increase was not statistically significant among any of the patient groups. Moreover, the cancer rate observed in case of adrenal lesions of more than 4 cm was at the lower limit of the range reported in the literature (from 2.4 to 31%) [19, 23–26]. In our opinion these data do not justify the systematic use of the open approach in all cases with large adrenal lesions, as suggested by some authors in literature [29–32]. In the present study, 214 patients out of 225 (95.1% of patients with adrenal lesions larger than 4 cm) benefited from the advantages of MIS.

Analyzing the operative time, and the conversion and complication rates, LA was found to be a safe and effective approach even for lesions larger than 4 cm in diameter, as previously reported by other authors [16, 27, 33, 34]. In eighteen cases (3.4%) of this series the surgeon decided to perform OA, although preoperatively there were no clear signs of malignancy (suggestive preoperative imaging), due to the suspicion of possible intraoperative technical problems. Only in four of these patients (0.8%) the adrenal lesions turned out to be malignant at definitive histology. In these patients the biggest concern is the risk of inadequate tumor removal or of capsular disruption of an unsuspected malignant tumor and the subsequent increase in the risk of local, peritoneal and port-site recurrences, as well as the possibility of intraoperative technical difficulties in tumor dissection. For these reasons, some authors recommend the open approach in these cases [21, 22, 29–32]. On the other hand, though, this results in a number of OA performed for benign adrenal lesions. Capsule ruptures with intraoperative tumor spillage did not occur in our study, and LA was performed with the same surgical and oncological safety in comparison to open surgery, so the questions we asked ourselves were: is it better to perform a laparotomy in all unverified cases of cancer, or is it better to start surgery laparoscopically and then convert it in case of evidence of malignancy? And then, analyzing the patients who underwent OA, is it more risky to perform LA for those patients who will turn out to have a malignant lesion, or is it more harmful to perform OA for patients who will prove to have a benign lesion? The guidelines do not give a clear direction on the type of approach to use in doubtful cases [5, 35, 36].

Our study suggests that LA should be preferred to OA even for lesions equal or larger than 4 cm, if there are no clear signs of malignancy, as long as an adequate MIS expertise is available on the surgeon’s part. Furthermore, LA has proven to be safe and effective even in cases that later turned out to be malignant at definitive histology.

This further supports the concept that size is not a valid and unique tool for preoperative assessment of adrenal malignancy [23–26, 37]. In fact, in the literature several cancer risk stratification algorithms for adrenal lesions are described, including other preoperative features as older age and male sex [23, 26], higher unenhanced CT attenuation [23, 26, 36], non-incidental mode of diagnosis [23], > 0.6 cm/year growth [26] and ^18^F-fluorodeoxyglucose positron emission tomography (PET) ratio > 1.5 [24].

Concerning management of adrenal incidentalomas (AIs), hormonal activity is an indication for surgery, as reported in the literature [19, 36], but it cannot be considered an indicator of malignancy. This is the reason why we decided not to exclude secreting tumors from our study. Moreover, the incidence of secreting lesions between malignant and benign histology in each group is not statistically significantly different.

Data reported in literature suggest that hormonally active lesions tend to be smaller than non-secreting ones, as they are symptomatic and discovered earlier [37]. This is in line with our study. In fact, although there are no statistically significant differences in the incidence of secreting tumors in benign and malignant lesions, in both groups hormonal activity is more frequent in lesions measuring less than 4 cm in diameter.

In the 2002 National Institutes of Health (NIH) consensus statement, the cancer risks by size in adrenal AIs were reported to be 2%, 6%, and 25% for lesions of < 4 cm, 4 to 6 cm, and > 6 cm, respectively [19]. Since then, in the literature there is no univocal consensus about the risk of cancer in adrenal lesions equal or larger than 4 cm in diameter [19, 23–25, 36–38]. Iñiguez-Ariza et al*.* reported a malignancy rate of 31% in a cohort of 705 patients with adrenal lesions of 4 cm or more in diameter [23]. In the study by Amodru et al*.*, in which 65 patients with non-secreting adrenal lesions of at least 4 cm were included, the risk of cancer was 20% [24]. The risk of malignancy reported by Cyranska-Chyrek et al*.* in a cohort of 2005 patients was 0.2% in lesions < 4 cm, 4.8% in the 4 to 6 cm group and 37.7% in lesions of more than 6 cm [25]. A recent retrospective review of 2219 patients by Kahramangil et al. described as the cancer risk per size in AIs is less than previously reported, probably because AI guidelines are based on data obtained with old-generation imaging [26]. Overall, adrenocortical carcinoma (ACC) incidence in AI was 1.7%, but stratified by size it was 0.1%, 2.4%, and 19.5% for AIs of less than 4 cm, 4 to 6 cm and more than 6 cm, respectively, and the optimal cut-off size for ACC in AI was 4.6 cm [26]. The rationale for these authors’ study was not far from ours and although the diagnosis of ACC is rare, it must be an early one, given the poor prognosis of metastatic ACC [26]. They recommend revising the AI guidelines considering a review of the importance of lesion size [26]. Therefore, it is mandatory to balance the risks and potential benefits when selecting patients for adrenalectomy [35–40].

As regards histologically proven malignant lesions, Xu Hu et al*.* compared MIS with OA in ACC in a recent meta-analysis [41]. They reported that although MIS was associated with earlier recurrence and more positive surgical margins, no statistically significant differences in survival were found [41]. On the contrary, Moreno et al*.* reported that LA was feasible for the treatment of selected patients with metastatic adrenal disease [42]. Most importantly, this surgical approach was associated with a significantly longer survival rate as compared to OA, probably in relation to a higher number of R0 resections obtained, although adrenal lesions in case of LA were significantly smaller [42].

Apart from the risk of malignancy in these patients, it is important to consider the intraoperative technical aspects. Some authors report that in case of large adrenal lesions, LA is associated with longer operative time as well as higher conversion and morbidity rates, but also this issue is still debated [27, 30, 31, 43, 44].

Regarding the operative time, some authors report a statistically longer operative time for adrenalectomy in case of adrenal lesions of more than 6 cm in diameter [27, 44, 45], but Rao et al*.* using a cut-off size of 4 cm did not report a statistically significantly longer operative time [46]. In the present series, an increase in the operative time was observed analysing each group according to increasing adrenal lesion size. However, this finding is not different from that reported in the literature [27, 44–46]. Moreover, statistically significant differences between benign and malignant lesions ware not observed in any groups. In terms of conversion rate, in the present study this increased with increasing adrenal lesion size, but conversion did not occur in any malignant case. A high conversion rate was observed only in group D (25%), while in the other groups this is similar to data reported in the literature, ranging from 0.5 to 21% [33, 34, 44, 47, 48]. Also, per group morbidity rates analysis observed in the present series are similar to previously published data [33, 44, 48, 49]. In the present series the complications that were observed did not occur in patients with malignant lesions at definitive histology.

The main limitations of the present study are its retrospective nature, a long-lasting study period, the small number of malignant lesions observed that may affect the statistical analysis and the lack of complete data on long-term follow up.

In conclusion, based on the present study, adrenal lesions measuring 4 cm or more in diameter are not a contraindication for laparoscopic surgery neither in terms of cancer risk nor considering conversion and morbidity rates, provided adequate MIS expertise is available, even if the operative time increases with increasing adrenal lesion diameter. In the five groups that are reported, the cancer risk ranges from 2.9 to 18.2% for adrenal lesions measuring from less than 4 cm to more than 10 cm. Hopefully, in the future a wider use of robotic MIS might improve the outcomes in these patients. Further prospective studies with a larger number of patients are required to draw more definitive conclusions.
